# Whole-Genome Resequencing of the VGSC Reveals the Evolutionary Mechanism of Pesticide Resistance in *Liriomyza trifolii* in Hainan

**DOI:** 10.3390/ijms27020732

**Published:** 2026-01-11

**Authors:** Linlin Yuan, Zhiyuan Lei, Junyi Zhang, Fen Li, Shaoying Wu

**Affiliations:** 1 School of Breeding and Multiplication (Sanya Institute of Breeding and Multiplication), Hainan University, Sanya 572025, China; yllyll0105@126.com (L.Y.); zyleimzy@163.com (Z.L.); zjy2024153708@163.com (J.Z.); 2 School of Tropical Agriculture and Forestry, Hainan University, Danzhou 571737, China

**Keywords:** *Liriomyza trifolii*, whole-genome resequencing, voltage-gated sodium channels, sSNPs, resistance

## Abstract

The extended application of pesticides has intensified the resistance problem in *Liriomyza trifolii* within Hainan Province. This study aimed to elucidate the underlying mechanisms contributing to the elevated resistance observed in this pest by employing Whole-Genome Re-sequencing (WGR) technology. Through the analysis and comparison of WGR data focusing on voltage-gated sodium channel (VGSC) from diverse regions and LT-S of *L. trifolii* in Hainan Province, we identified a total of six nonsynonymous single nucleotide polymorphisms (nsSNPs) and thirty-one synonymous single nucleotide polymorphisms (sSNPs) in five wild populations MY, TS, DA, TY, and JY. Among the six nsSNPs, three (PyR1: M918T, L1014F, and PyR2: T933I) have been confirmed as linked to pyrethroid resistance, while one (D IVS6: V1845I) was associated with resistance to indoxacarb. Moreover, the frequency of these four mutations generally increases with decreasing latitude. Additionally, under sustained pesticide selection pressure, *L. trifolii* exhibits an evolutionary pattern characterized by a dN/dS ratio (nsSNP/sSNP = 6/31 ≈ 0.19) of less than 1. Among the 31 sSNPs that held an absolute quantitative advantage, the highest occurrence frequency reached 94.44% (G2033: JY), and this sSNP occurred in all populations. In contrast, among a limited number of 6 nsSNPs, the highest occurrence frequency attained 100% (L1014F: all populations). This study substantiates that the elevated resistance observed in *L. trifolii* within Hainan Province can be ascribed to the presence of four nsSNPs-M922T, T933I, L1018F, and V1845I in their VGSC. Furthermore, the emergence of cross-resistance between pyrethroids and indoxacarb has been identified. This research offers a novel theoretical foundation for future investigations into the resistance mechanisms of *L. trifolii*.

## 1. Introduction

*Liriomyza trifolii* (Burgess), an invasive pest species in China, initiated its global dissemination from Florida, USA [1,2]. Currently, the distribution of *L. trifolii* is primarily concentrated in the southeastern coastal regions of China, encompassing Hainan, Guangdong, Guangxi, Shanghai, Fujian, Zhejiang, Jiangsu, and Taiwan. *L. trifolii* exhibits a broad host range, impacting crops such as legumes, Solanaceae, Cucurbitaceae, and cruciferous plants [3], with documented evidence of host range expansion [4]. This pest is distinguished by its diminutive size, high reproductive capacity, brief development cycle, and rapid spread dispersal, frequently resulting in overlapping generations, which predisposes it to outbreaks and substantial agricultural damage [5]. In warmer regions, *L. trifolii* can cause continuous damage throughout the year due to overlapping generations.

Hainan Province, located in southern China, functions as the principal distribution hub for winter fruits and vegetables in the region, with an annual cultivation area exceeding 60,000 hectares. This represents a significant challenge for agriculture in Hainan. The year-round cowpea cultivation creates highly favorable conditions for *L. trifolii* populations expansion, essentially providing a continuous host and breeding ground. This, combined with the extended and frequent pesticide exposure, creates a classic scenario for the rapid development of pesticide resistance. Currently, both domestically and internationally, chemical pesticides remain the primary method for controlling *L. trifolii*. The widespread and excessive application of pesticides has led to a continuous rise in wild population resistance.

Pyrethroids are a significant class of neurotoxic pesticides [6]. Their mode of action involves disrupting the function of the insect’s peripheral and central nervous systems [7]. Specifically, pyrethroids target voltage-gated sodium channels (VGSC) as their main site of activity [8]. Mutations at VGSC sites can lead to knockdown resistance (kdr), the primary mechanism of pyrethroid resistance, by reducing the pesticide’s affinity for its target [9,10]. This same VGSC target is also exploited by other insecticides, including indoxacarb and flufenidin [11,12,13,14]. VGSC comprises four homologous internal domains (I–IV), each containing six transmembrane α-helices (S1–S6) (Figure S1). These domains are interconnected by intracellular linkers, while the helices are linked by either intracellular or extracellular loops [15]. The homologous channel model suggests that pyrethroids and VGSC possess two distinct binding regions: Pyrethroid Resistance site 1 (PyR1) (II L45-II S5-III S6) [16] and Pyrethroid Resistance site 2 (PyR2) (I L45-I S5-I S6-IIS6) [17]. Research has extensively focused on mutations within these binding regions (PyR1 and PyR2), as such mutations are likely to diminish the sensitivity of insect VGSC to pyrethroids, thereby contributing to increased insect resistance to these pyrethroids.

Whole Genome Re-sequencing (WGR) is a process that swiftly and accurately identifies all the “differences” between a specific individual and the “standard map” (reference genome) based on the existing “map” information. WGR has emerged as a prominent application in Next-Generation Sequencing (NGS). Relevant studies have conducted genotyping on populations exhibiting varying levels of pyrethroid resistance in northern Brazil, focusing on genetic polymorphisms within the genomes of *Aedes aegypti* with distinct characteristics. The allele frequencies across the entire genome were assessed using SNP chips, leading to the identification of single nucleotide polymorphisms (SNPs) directly associated with resistance, as well as one superior SNP pair [18]. WGR of *Spodoptera frugiperda* from the Indochinese Peninsula to northern China (2019–2023) identified eight stable genomic markers (six SNPs, two InDels) that clearly distinguish eastern and western populations. This work established a reliable molecular toolkit for tracing the origin of individual moths [19].

This study employed WGR to investigate the underlying mechanisms of high resistance in *L. trifolii* populations from Hainan. By comparing the VGSC across various geographic strains and a susceptible reference, four critical VGSC mutations were identified. These mutations confer high levels of resistance and cross-resistance to pyrethroids and indoxacarb, offering crucial molecular targets for future resistance management strategies.

## 2. Results

### 2.1. Complete Genomic Information of VGSC in L. trifolii

**Figure 1:**
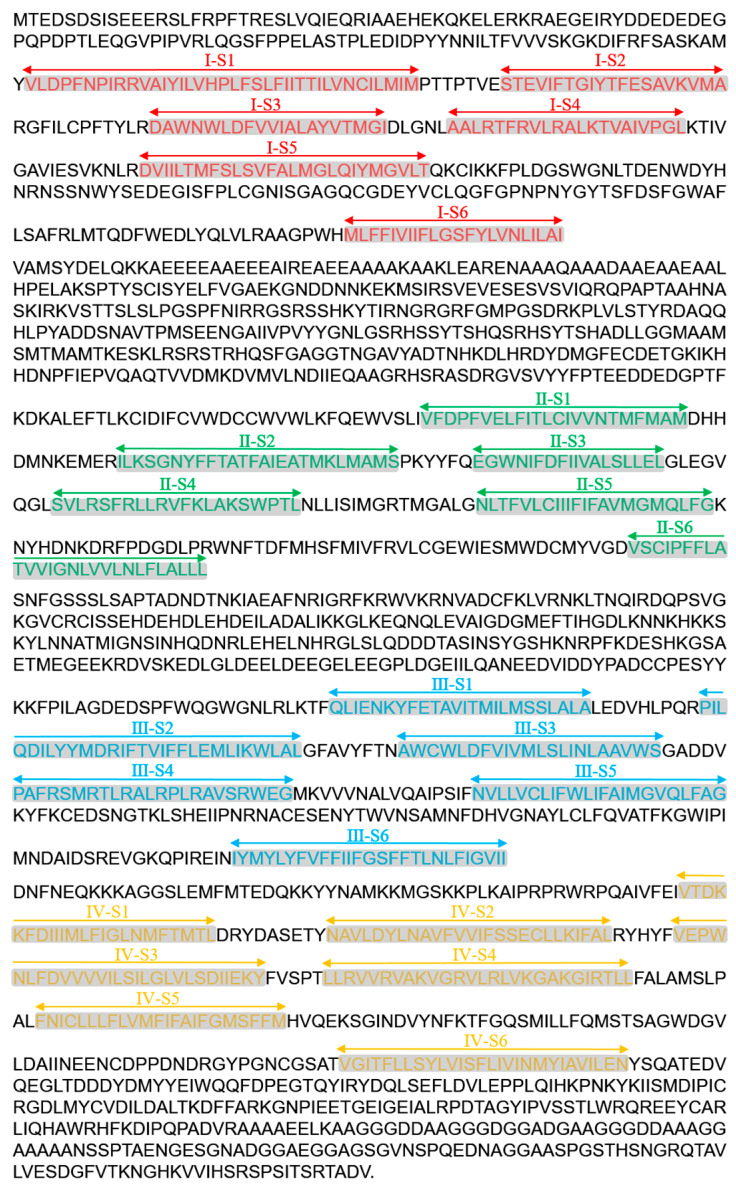
Amino acid information map of the complete CDS region, including four domains and each helical fragment of the VGSC from the sensitive strain of *L. trifolii*.

Following the analysis of first-generation sequencing results, the complete genomic sequence of the VGSC of *L. trifolii* was determined to be 36,774 base pairs (bp). This sequence comprises specific lengths for Domain I (6696 bp), Domain II (10,162 bp), Domain III (11,025 bp), and Domain IV (8891 bp). Within the entire genomic sequence, the intron and coding sequence (CDS) lengths are 30,360 bp and 6414 bp, respectively (Figure S2). The focused 6414 bp CDS segment contains a total of 2138 amino acids, including the stop codon. This segment encodes 415, 614, 531, and 578 amino acids for Domain I, Domain II, Domain III, and Domain IV, respectively (Figure 1).

### 2.2. Information of Exonic Synonymous sSNP in L. trifolii

**Figure 2:**
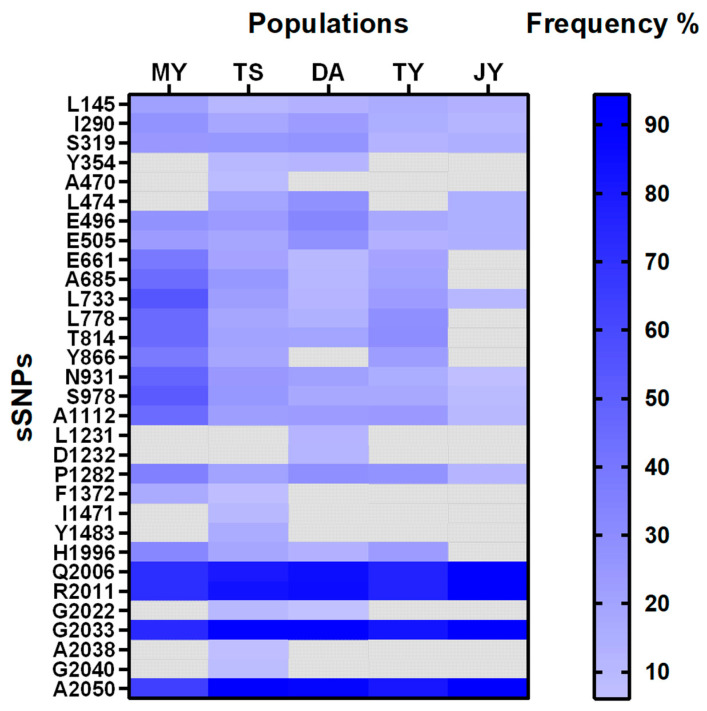
Heat maps of the occurrence and frequency distribution of 31 sSNPs in five populations.

Based on codon degeneracy, the 31 identified sSNPs were classified as L145L, I290I, S319S, Y354Y, A470A, L474L, E496E, E505E, E661E, A685A, L733L, L778L, T814T, Y866Y, N931N, S978S, A1112A, L1231L, D1232D, P1282P, F1372F, I1471I, Y1483Y, H1996H, Q2006Q, R2011R, G2022G, G2033G, A2038A, G2040G, and A2050A. These sSNPs are primarily distributed across the following regions: “D I-S1”, “D I-S56-L”, “D I-II-L”, “D II-S1”, “D II-S23-L”, “D II-S5”, “D II-S56-L”, “D II-III-L”, “D III-S3”, “D III-S56-L” and “D IV < L”. The positions of these sSNPs within the VGSC, along with the nucleotide alterations and occurrence frequencies, are detailed in Table 1. Notably, 14 sSNPs were found in all five wild populations (MY, TS, DA, JY, TY), specifically L145L, I290I, S319S, E496E, E505E, L733L, N931N, S978S, A1112A, P1282P, Q2006Q, R2011R, G2033G, and A2050A. Among these, Q2006Q exhibited the highest occurrence rate at 94.31%, while N931N had the lowest at 7.08%. Furthermore, both the highest and lowest frequencies were observed in the JY population. Of the 31 sSNPs, A470A, L1231L, D1232D, I1471I, Y1483Y, A2038A, and G2040G were found exclusively in a single population, with frequencies of 8.57% (TS), 11.76% (DA), 12.24% (DA), 10.10% (TS), 16.19% (TS), 7.04% (TS), and 8.33% (TS), respectively (Figure 2).

### 2.3. Information of Exonic nsSNP in L. trifolii

**Figure 3:**
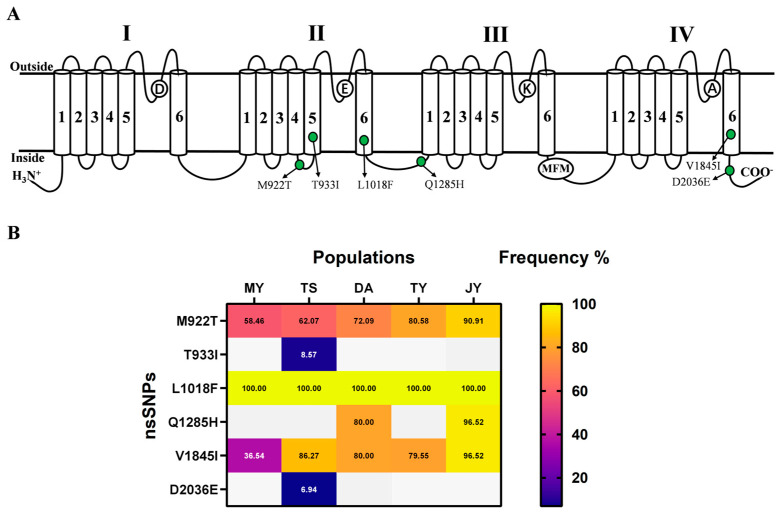
The positions of six nsSNPs on the VGSC topological map. The (**A**) occurrence and (**B**) frequency distribution heat maps in the five populations. “1”, “2”, “3”, “4”, “5” and “6” represent six “Hydrophobic transmembrane helix”; “D”, “E”, “K” and “A” represent ion-selective filters; green dots represent “mutations”.

After conducting multiple comparisons and analyses of the CDS region in the genome resequencing data, our results identified 6 nsSNPs in the VGSC of *L. trifolii*: M922T (918), T933I, L1018F (1014), Q1285H, V1845I (1851), and D2036E. These nsSNPs are primarily situated in specific regions of the VGSC: D II-S45-L (M922T), D II-S5 (T933I), D II-S6 (L1018F), D II-III-L (Q1285H), D IV-S6 (V1845I), and D IV < L (D2036E) (Figure 3A). The positions, nucleotide alterations, and frequencies of these nsSNPs in the VGSC are detailed in Table 2. Notably, 3 nsSNPs, namely M922T, L1018F, and V1845I, were present across all five wild populations (MY, TS, DA, JY, TY). Among these, L1018F exhibited the highest occurrence rate of 100.00%, which was consistent across all five populations. M922T exhibits the highest incidence rate in JY (90.91%) and the lowest in MY (58.46%). Similarly, V1845I shows the highest frequency in JY (96.52%) and the lowest in MY (36.54%), mirroring the pattern observed for M922T. T933I and D2036E are unique to the TS population among the 6 nsSNPs. The frequencies of Q1285H and V1845I in the DA and JY populations are identical at 80.00% and 96.52%, respectively. Figure 3B illustrates the frequency comparison of various nsSNPs across populations in a north-to-south order based on decreasing latitude.

## 3. Discussion

The high level of resistance and unique reproductive capabilities are the primary factors contributing to *L. trifolii* ‘s status as a significant pest [20]. For decades, deltamethrin has been extensively employed to manage *L. trifolii*. However, subsequent studies have demonstrated that deltamethrin is no longer effective against this pest [21]. In 1979, permethrin was introduced in California, United States, for the control of *L. trifolii*. Unfortunately, during the period of 1985–1987, its effectiveness diminished due to the pest’s increased resistance [22,23]. Additionally, during the period from 1989 to 1991, *L. trifolii* exhibited varying degrees of resistance to permethrin, deltamethrin, and several other insecticides [24,25]. The sustainable management of crop pests in agricultural fields has been significantly compromised by the emergence of pesticide resistance [26], a challenge that is particularly pronounced in tropical regions [27].

For the identified nsSNPs, M918T (a nsSNP of M922T in *L. trifolii*), conferred exceptionally high resistance to deltamethrin in rat VGSC [28]. Furthermore, in 2011 and 2012, M918T was confirmed to be linked to resistance against pyrethroid insecticides in *Tetranychus evansi* and *Aphis gossypii* [29]. The M918L variant at the same position was identified in *Hyelella azteca* [30]. Additionally, M918V was discovered in *Bemisia tabaci* [31]. When examined in *Xenopus* oocytes, various substitutions can confer differing levels of resistance. Distinct mutant VGSC offer varying degrees of protection against type I and type II pyrethroids [16]. This phenomenon has been observed at the M918 (T/L/V) site, where the M918T substitution conferred the highest level of protection against permethrin and deltamethrin [28]. In this study, only the M918T variant was identified in *L. trifolii*, and the mutation frequency exhibited a gradual increase as latitude decreased, ultimately reaching a maximum of 90.91%. This finding suggests that *L. trifolii* populations in Hainan Province may have developed a high level of resistance to pyrethroids. This could partially explain the widespread detection of the potent M918T substitution across different populations and its consistently high frequency. The observed correlation provides important clues for further elucidating the underlying resistance mechanisms. Furthermore, the data show that the occurrence frequency of these mutations is correlated with the increase in temperature in low-latitude regions. This trend may be related to the shortened generation cycle of *L. trifolii* under higher temperatures, and the shortening of the generation cycle might facilitate the accumulation of insecticide resistance in its offspring.

Among the six nsSNPs identified in this study, mutations consistently linked to pyrethroid resistance include M918 (mutate to T, L, or V), as well as L1014 (mutate to F, C, H, S, or W) (L1018F in *L. trifolii*) and T933 (mutate to I, C, or V). Notably, L1014F is situated in PyR1, similar to M918, whereas T933 is found in PyR2. Therefore, M918 and L1014F within PyR1 will be analyzed in our results firstly, followed by a discussion of T933 located in PyR2.

The first mutation identified and confirmed as kdr-related was L1014F in house fly (L1018F in *L. trifolii*) [32]. L1014F provides variable levels of protection to Type I or Type II pyrethroids or DDT [33]. Prior research has demonstrated that the L1014F single mutation confers resistance to pyrethroids in various species, including *Anopheles stephensi* [34], *A. gossypii* [29], *Blattella germanica* [8,35], *Ctenocephalides felis* [36], *Cydia pomonella* [37], *Frankliniella occidentalis* [38], *Leptinotarsa decemlineata* [39], *Liriomyza huidobrensis* [40], *Meligethes aeneus* [41], *Musca domestica* [35,42], *Sitobion avenae* [43], *Triatoma infestans* [44] and *Liriomyza sativae* [40], etc. The variants L1014S, L1014H, L1014C, and L1014W were identified at position 1014 [40,45,46]. In contrast, only one mutant type, L1018F (consistent to L1014F), was observed in *L. trifolii*, exhibiting a frequency of occurrence of 100% across all five populations. These results point to the possibility that *L. trifolii* in Hainan Province has evolved a considerable degree of pyrethroid resistance. This presents a plausible explanation for the persistent survival of these populations under standard insecticide application.

The T933I, corresponding to the amino acid number in *L. trifolii*, has been linked to resistance against pyrethroids in various pests, including *Thrips palmi* [47], *Trialeurodes vaporariorum* [48], *L. decemlineata* [49] and *Thrips tabaci* [50]. Other single mutations at this position include T933C and T933V. Additionally, T929C was identified in *F. occidentalis* [38], while T933V has been observed in *C. felis* [36] and *F. occidentalis* [38]. In this study, only the T933I mutation was identified, and this mutation only occurred at a relatively low frequency of 8.57% in the TS. The outcome could be attributed to TS being a classic “mountain city” enclosed by mountains from all directions, leading to reproductive isolation or minimal gene flow with other populations. In small and secluded populations, particular mutations are prone to enhancing survival chances, despite lacking an immediate advantage. The T933I evidently confers a survival advantage special to the environmental challenges encountered by this population, including the intensity and frequency of local pesticide use and the cold temperatures prevalent in mountainous regions.

Prior research has demonstrated that *L. trifolii* populations with avermectin tolerance display the highest resistance to cypermethrin, a type II pyrethroid [51]. Consequently, cross-resistance between pyrethroids and other insecticides has surfaced in *L. trifolii*. Moreover, in the Sanya City wild of Hainan Province, China, in 2022, the sensitivity of second-instar *L. trifolii* larvae to indoxacarb decreased by 776.17 times, with resistance to avermectin exhibiting a consistent upward trajectory [52]. One of the nsSNP identified in this study, V1845I (amino acid position in *L. trifolii*), has been confirmed to be associated with resistance to indoxacarb in *Plutella xylostella* [12]. Subsequently, V1845I resistance-associated mutation was also detected in *Tuta absoluta* [11]. Furthermore, this mutation has been reported in *L. trifolii* [27]. Research has confirmed, using a two-electrode voltage clamp system, that the V1845I mutant channel are more resistant to indoxacarb, DCJW and metaflumizone than wild type channels [10]. Moreover, the resistance mutation V1845I of the VGSC blocker insecticides (SCBIs) was functionally validated in Drosophila through molecular simulation and genomic engineering [53]. The data indicate a correlation in Hainan *L. trifolii* populations between exceptionally high pyrethroid resistance and a concurrent, relatively elevated resistance to indoxacarb, which is consistent with the presence of cross-resistance mechanisms. The findings of this study indicate that as latitude decreases, the mutation frequency of V1845I exhibits a general upward trend, peaking at 96.52%. The mutation frequency in the TS region is notably higher than that observed in high-latitude areas. This discrepancy may be attributed to the widespread application of indoxacarb in the agricultural practices of the TS region.

Moreover, research has demonstrated that double mutations, in contrast to single mutations, nearly abolish the sensitivity of *Drosophila melanogaster* VGSC to deltamethrin [28,39]. This finding suggests that the impact of the double mutation surpasses that of the single mutation. Notably, the double mutant Q1285H + V1845I exhibited identical frequency in the DA and JY populations, at 80.00% and 96.52%, respectively. Due to the sequencing methods and characteristics of WGR, it remains uncertain whether Q1285H + V1845I represents a genuine tandem double mutation. Nevertheless, based on frequency analysis, there is a likelihood of the occurrence of the double mutant Q1285H + V1845I. Whether Q1285H + V1845I is a double mutation requires further verification through haplotype analysis. The functional implications of this dual process necessitate validation through electrophysiology-related experiments in future investigations. The D2036E variant identified in this study is located in the non-domain region of the VGSC of *L. trifolii*. Further investigation is necessary to determine its potential association with drug resistance in this species. It is important to note that while the identified mutations in our WGR data have been previously associated with insecticide resistance in related species, we did not perform direct bioassays to confirm the resistant phenotype of *L. trifolii* populations used in this study. Therefore, the functional link between these specific mutations and resistance in our sampled populations remains inferential.

In addition to the numerous nsSNPs analyzed above, our results identified a substantial number of sSNPs in the VGSC of *L. trifolii*, with their prevalence significantly exceeding that of nsSNPs. Furthermore, accurately quantifying the number of nsSNPs and sSNPs, can also determine whether a small-scale population is prone to the accumulation of harmful variations, potentially resulting in uncontrolled mutations or even species extinction and outbreaks [54,55]. In evolutionary research, the ratio of nsSNP to sSNP (dN/dS) serves as a crucial indicator for assessing whether a gene is subject to natural selection pressure. When natural selection facilitates alterations in protein sequences, the dN/dS ratio is anticipated to exceed 1. Conversely, when natural selection restricts changes in proteins, the ratio falls below 1 [56,57,58]. This interpretation of dN/dS is further substantiated by theoretical analyses of the relationship between dN/dS statistics and potential selection pressure within the Wright-Fisher model [59]. In this study, the dN/dS ratios observed across all five *L. trifolii* populations were below 1. It is important to note that while a dN/dS < 1 is a classic signature of purifying selection, its interpretation from population-level SNP data requires caution. With this caveat in mind, our results suggest several possible evolutionary implications. First, the low dN/dS ratio may indicate that a majority of deleterious nsSNPs in the VGSC are effectively purged from the population. Individuals carrying such mutations, which are likely to disrupt protein structure and function, probably experience reduced fitness, leading to their selective removal over time. Next, this pattern underscores the critical functional importance of the VGSC. Its protein sequence appears to be under strong selective constraint, implying that most random amino acid changes are detrimental to survival and reproduction. Consequently, the gene exhibits high evolutionary conservation. Furthermore, the persistence of any nsSNP that rises to detectable frequency could represent a site of potential adaptive significance. In the context of intense insecticide pressure, such variants might confer a selective advantage. However, distinguishing such adaptive variants from nearly neutral polymorphisms requires complementary population genetic analyses. Finally, the consistently low dN/dS ratio across distinct populations supports the hypothesis that the core functional domains of the VGSC have remained highly conserved over a prolonged evolutionary period, with strong selective pressure to maintain ancestral protein function. Given the unique environmental pressures in Hainan Province, including persistent high temperatures, humidity, and continuous pesticide application, the evolutionary dynamics of the VGSC and the functional role of specific nsSNPs in *L. trifolii* worth further research through integrated population genomic and functional verification approaches.

The mutations identified in this study represent the first report in *L. trifolii* of Hainan. Furthermore, the potential correlation between latitude and mutation frequency constitutes a novel finding in *L. trifolii*. Additionally, we are the first to propose that *L. trifolii* in the Hainan region exhibits cross-resistance to pyrethroids and indoxacarb. In conclusion, the key mutations identified in this study, including M918T, L1014F, T933I, and V1845I, serve as effective molecular markers for monitoring *L. trifolii* resistance in the Hainan region. Implementing a rotation of insecticides with different mechanisms in areas with high mutation frequencies can delay the development of resistance, prolong the efficacy of existing insecticides, and ensure the safe production of Hainan’s vegetable industry.

## 4. Materials and Methods

### 4.1. Insects Collection

**Figure 4:**
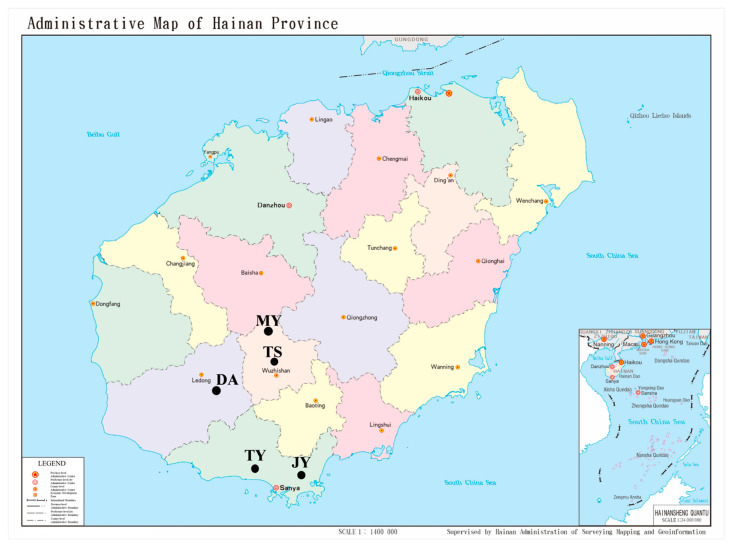
Origin information of population samples for whole-genome resequencing (WGR).

The sensitive strain of *L. trifolii* (LT-S) used in this study was provided by Professor Yuzhou Du of Yangzhou University. This strain has been maintained in the laboratory without exposure to insecticides for over two decades. The sensitivity of LT-S to indoxcarb was previously determined to be 0.822 mg·L^−1^ [27]. The entire life cycle of the insects was raised at a temperature of 26 ± 1 °C, the humidity should be maintained at 70% ± 5% and L:D = 16 h:8 h. The adult insects laid eggs and the larvae were raised using cultivated bean seedlings (Baijiali cowpea seeds, USA) that were not exposed to any pesticides. Wild populations selected for WGR were gathered from five locations: Tian-Ya (TY: N 18.31, E 109.48) and Ji-Yang (JY: N 18.29, E 109.54) in Sanya, Mao-Yang (MY: N 18.94, E 109.51) and Tong-Shi (TS: N 18.79, E 109.52) in Wuzhishan, and Da-An in Ledong (DA: N 18.47, E 108.90) (Figure 4).

### 4.2. Acquisition of Complete Genomic of VGSC in L. trifolii and Sequencing WGR

Genomic DNA extracted from LT-S was single-headed and processed using the Universal Genomic DNA Extraction Kit (Beijing Solarbio Science & Technology Co., Ltd., Beijing, China). Segmented gene amplification on the complete genomic sequence of VGSC of *L. trifolii* was conducted utilizing the Phanta-Max Super-Fidelity DNA Polymerase kit (Vazyme Biotech Co., Ltd., Nanjing, China). By integrating the genomic data of the VGSC from *L. trifolii* (GenBank: GCA_001014935.1), primers were formulated for cloning to acquire the complete genome sequence of the susceptible strain of the VGSC from *L. trifolii*. Details of the primers and annealing temperatures employed for fragment amplification can be found in Table 3. Subsequently, PCR products were submitted to Tsingke Biotechnology Co., Ltd. (Beijing, China) for first-generation sequencing, and the DNASTAR Lasergene 11 Core Suite software was utilized to assemble the complete genomic sequence of VGSC.

Large number of insects from the LT-S cultivated indoors and wild populations were sent to the Beijing Genomics Institute (BGI Genomics Co., Ltd., Beijing, China) for WGR. Genomic data analysis was conducted utilizing the GeneAn cloud platform (https://www.bgitechsolutions.com/technologies/263, accessed on 4 December 2025). The sample size for each group of WGR experiments was all greater than 250 individual specimens of *L. trifolii*. For the sensitive strains, after being paired with one female and one male (F1), the pupae of the next generation (F2) are collected and directly sequenced. The wild populations do not undergo multiple generations of indoor breeding. Instead, samples are directly collected from the wild and sequenced. The original sequencing data underwent rigorous quality control. Initial quality assessment was performed using FastQC (v0.11.9). Following quality control, the average Q30 base ratio across all samples exceeded 85%. Coverage depth was calculated using SAMtools (v1.15). The results indicated that the average genome-wide coverage depth for all samples surpassed 20 times, with over 95% of genomes exhibiting a coverage depth of at least 10 times, thereby ensuring the reliability of subsequent variant detection. In this study, the coverage depth in the regions containing the VGSC gene consistently exceeded the average level of the entire genome.

### 4.3. Analysis and Acquisition of sSNP and nsSNP in VGSC of L. trifolii

The genomic fragment sequences of the VGSC from the LT-S strain were concatenated to derive the complete full-length genomic sequence of the VGSC of *L. trifolii*. This complete VGSC genomic sequence will be annotated according to the Coding Sequence (CDS) (GenBank: MT648287.1), and the nucleotide position information of the intron and exon segments will be scrutinized. Mutants situated in the exon region were filtered from the outcomes of WGR using the aforementioned nucleotide position information. Subsequently, by correlating the codon information of each mutation in the exon with the CDS position, our results classified the mutations as synonymous single nucleotide polymorphism (sSNP) or non-synonymous single nucleotide polymorphism (nsSNP), determined their positions on the VGSC, and computed their frequencies of occurrence. The approach for distinguishing between sSNPs and nsSNPs is illustrated in Figure S3, where the section highlighted in red elucidates the method for identifying sSNPs as discussed in this study.

## Figures and Tables

**Table 1:** Positions, variations and frequencies of 31 sSNPs in the VGSC of *L. trifolii*.

Position ^d^	sSNPs	Nucleotide Alteration	Frequency/% ^e(f,g)^				
			MY	TS	DA	TY	JY
D I-S1	L145	TTA > TTG	21.33 (59, 16)	11.36 (78, 10)	13.11 (53, 8)	16.22 (62, 12)	13.11 (53, 8)
D I-S56-L	I290	ATT > ATC	27.05 (89, 33)	18.03 (100, 22)	23.08 (60, 18)	15.65 (97, 18)	12.07 (102, 14)
	S319	TCT > TCA	24.77 (82, 27)	25.00 (72, 24)	26.47 (50, 18)	12.90 (81, 12)	14.00 (86, 14)
	Y354	TAT > TAC	-	9.89 (82, 9)	11.96 (81, 11)	-	-
D I-II-L	A470	GCG > GCT	-	8.57 (64, 6)	-	-	-
	L474	TTA > TTG	-	19.70 (53, 13)	28.24 (61, 24)	-	14.55 (94, 16)
	E496	GAG > GAA	27.27 (48, 18)	23.91 (35, 11)	32.94 (57, 28)	17.72 (65, 14)	14.46 (71, 12)
	E505	GAA > GAG	22.95 (47, 14)	18.75 (39, 9)	28.57 (55, 22)	13.33 (52, 8)	14.08 (61, 10)
	E661	GAG > GAA	39.18 (59, 38)	19.18 (59, 14)	10.00 (81, 9)	18.95 (77, 18)	-
	A685	GCC > GCA	44.33 (54, 43)	25.00 (51, 17)	11.24 (79, 10)	20.93 (68, 18)	-
	L733	TTG > TTA	54.22 (38, 45)	21.95 (64, 18)	11.59 (61, 8)	22.69 (92, 27)	10.24 (114, 13)
	L778	CTC > CTT	45.53 (67, 56)	18.63 (83, 19)	13.49 (109, 17)	28.91 (91, 37)	-
D II-S1	T814	ACA > ACG	45.28 (58, 48)	20.00 (80, 20)	19.82 (89, 22)	29.91 (75, 32)	-
D II-S23-L	Y866	TAC > TAT	38.46 (56, 35)	18.68 (74, 17)	-	22.23 (70, 20)	-
D II-S5	N931	AAC > AAT	48.00 (39, 36)	24.64 (52, 17)	21.43 (66, 18)	16.16 (83, 16)	7.08 (105, 8)
D II-S56-L	S978	TCT > TCG	52.87 (41, 46)	25.00 (54, 18)	17.71 (79, 17)	18.00 (82, 18)	9.16 (119, 12)
D II-III-L	A1112	GCT > GCA	44.87 (43, 35)	21.88 (50, 14)	23.38 (59, 18)	24.44 (68, 22)	9.76 (74, 8)
	L1231	CTA > CTT	-	-	11.76 (90, 12)	-	-
	D1232	GAT > GAC	-	-	12.24 (86, 12)	-	-
	P1282	CCT > CCG	35.71 (72, 40)	20.72 (88, 23)	28.70 (77, 31)	27.17 (67, 25)	11.72 (113, 15)
D III-S3	F1372	TTT > TTC	16.67 (30, 6)	7.50 (37, 3)	-	-	-
D III-S56-L	I1471	ATC > ATT	-	10.10 (89, 10)	-	-	-
	Y1483	TAT > TAC	-	16.19 (88, 17)	-	-	-
D IV < L	H1996	CAC > CAT	32.43 (75, 36)	18.52 (66, 15)	13.21 (92, 14)	23.15 (83, 25)	-
	Q2006	CAA > CAG	72.32 (31, 81)	80.28 (14, 57)	85.15 (15, 86)	76.77 (23, 76)	94.31 (7, 116)
	R2011	CGA > CGT	72.22 (30, 78)	82.54 (11, 52)	85.29 (15, 87)	76.84 (22, 73)	93.97 (7, 109)
	G2022	GGT > GGC	-	10.00 (54, 6)	6.06 (93, 6)	-	-
	G2033	GGG > GGT	73.63 (24, 67)	91.94 (5, 57)	91.86 (7, 79)	81.94 (13, 59)	94.44 (6, 102)
	A2038	GCG > GCT	-	7.04 (66, 5)	-	-	-
	G2040	GGA > GGT	-	8.33 (66, 6)	-	-	-
	A2050	GCC > GCT	64.41 (21, 38)	94.29 (4, 66)	88.57 (8, 62)	81.36 (11, 48)	93.40 (7, 99)

Note: The meanings represented by each symbol in position “^d^” are as follows: “<” indicates being located “behind” a certain position; “>” indicates the transformation from one codon to another; “D” represents “Domain”; “L” represents “Linker”; “S” represents “Helix Segment”; “I”, “II”, “III” and “IV” represent the naming of four domains, respectively. “^f^” represents the number of times that no nucleotide change occurred at a specific sSNP position during the WGR, while “^g^” represents the number of times that a nucleotide change occurred at that sSNP position. The calculation method for “^e^” is: e = g/(f + g).

**Table 2:** Positions, variations and frequencies of 6 nsSNPs in the VGSC of *L. trifolii*.

Position ^d^	nsSNPs	Nucleotide Alteration	Frequency/% ^h(i,j)^				
			MY	TS	DA	TY	JY
D II-S45-L	M922T	ATG > ACG	58.46 (27, 38)	62.07 (22, 36)	72.09 (24, 62)	80.58 (20, 83)	90.91 (11, 110)
D II-S5	T933I	ACA > ATA	-	8.57 (64, 6)	-	-	-
D II-S6	L1018F	CTT > TTT	100.00 (0, 64)	100.00 (0, 71)	100.00 (0, 90)	100.00 (0, 102)	100.00 (0, 126)
D II-III-L	Q1285H	CAA > CAC	-	-	80.00 (18, 72)	-	96.52 (4, 111)
D IV-S6	V1845I	GTT > ATA	36.54 (99, 57)	86.27 (14, 88)	80.00 (18, 72)	79.55 (18, 70)	96.52 (4, 111)
D IV < L	D2036E	GAC > GAA	-	6.94 (67, 5)	-	-	-

Note: The meanings represented by each symbol in position “^d^” are as follows: “<” indicates being located “behind” a certain position; “>” indicates the transformation from one codon to another; “D” represents “Domain”; “L” represents “Linker”; “S” represents “Helix Segment”; “II”, “III” and “IV” represent the naming of three domains, respectively. “^i^” represents the number of times that no nucleotide change occurred at a specific nsSNP position during the WGR, while “^j^” represents the number of times that a nucleotide change occurred at that nsSNP position. The calculation method for “^h^” is: h = j/(i + j).

**Table 3:** Primers Information and annealing temperatures for VGSC in *L. trifolii.*

Primers	Sequence	Product Length ^a(b,c)^ (bp)	Annealing Temperature (°C)
LT-1F	ATGACAGAAGATTCCGACTCGA	288 (215, 73)	47.5
LT-1R	TTCCGGCGGGAAGCTGCCCTGCAAT		
LT-2F	GGTCCACAACCGGATCCTAC	1671 (188, 1483)	39.0
LT-2R	TGGCTACACGACGTATTGGA		
LT-3F	TCCAATACGTCGTGTAGCCA	2811 (236, 2575)	41.5
LT-3R	AAGTCCAGCCAATTCCATGCA		
LT-4F	GTGATGGCACGAGGTTTCAT	1982 (713, 1269)	40.0
LT-4R	TCGTCATACGACATGGCAAC		
LT-5F	CGAATTGCAAAAGAAAGCCGA	3701 (565, 3136)	41.5
LT-5R	TTCAGACATCGGTGTGACGG		
LT-6F	CCGTCACACCGATGTCTGAA	4563 (610, 3953)	44.5
LT-6R	TCGAAGACAATTAATGACACCCA		
LT-7F	TGGGTGTCATTAATTGTCTTCGA	1898 (697, 1201)	45.0
LT-7R	TGGACAAAAGCAAGGCCAAG		
LT-8F	TGGCCTTGCTTTTGTCCAATT	4357 (796, 3561)	42.0
LT-8R	AAATTGCCCCATCCTTGCCA		
LT-9F	GGCAATTTACGACTGAAAACTTTTCA	1008 (225, 783)	49.0
LT-9R	ATACACCGCAAACCCGAGAG		
LT-10F	CTGCCGCAAAGACCCATACT	4363 (301, 4062)	39.0
LT-10R	GAACCAGCGCATTAACGACG		
LT-11F	CGTCGTTAATGCGCTGGTTC	4544 (584, 3960)	39.5
LT-11R	ACTATTGCTTGTGGTCGCCA		
LT-12F	CGACCACAAGCAATAGTTTTTGA	3583 (327, 3256)	44.5
LT-12R	CAGCACACGACCGACTTTTG		
LT-13F	GTGTGGTACGTGTGGCAAAA	2247 (1274, 973)	38.5
LT-13R	TCAGACATCCGCCGTGCGTG		

Note: “^a^” represents the total length of the PCR (Polymerase Chain Reaction) product, “^b^” is the length of exons in the total length, and “^c^” is the length of introns.

## Data Availability

For confidentiality reasons, the original data supporting the results of this study have not been stored publicly. However, processed data may be obtained from the corresponding author upon reasonable request, provided that ethical and confidentiality agreements are upheld.

## Supplementary Materials

The following supporting information can be downloaded at: https://www.mdpi.com/article/10.3390/ijms27020732/s1.
