# Supplementary figures and images for: Whole-Genome Resequencing of the VGSC Reveals the Evolutionary Mechanism of Pesticide Resistance in Liriomyza trifolii in Hainan
Source: Int J Mol Sci. 2026 Jan 11;27(2):732. doi: 10.3390/ijms27020732 (PMC12840826; doi:10.3390/ijms27020732)

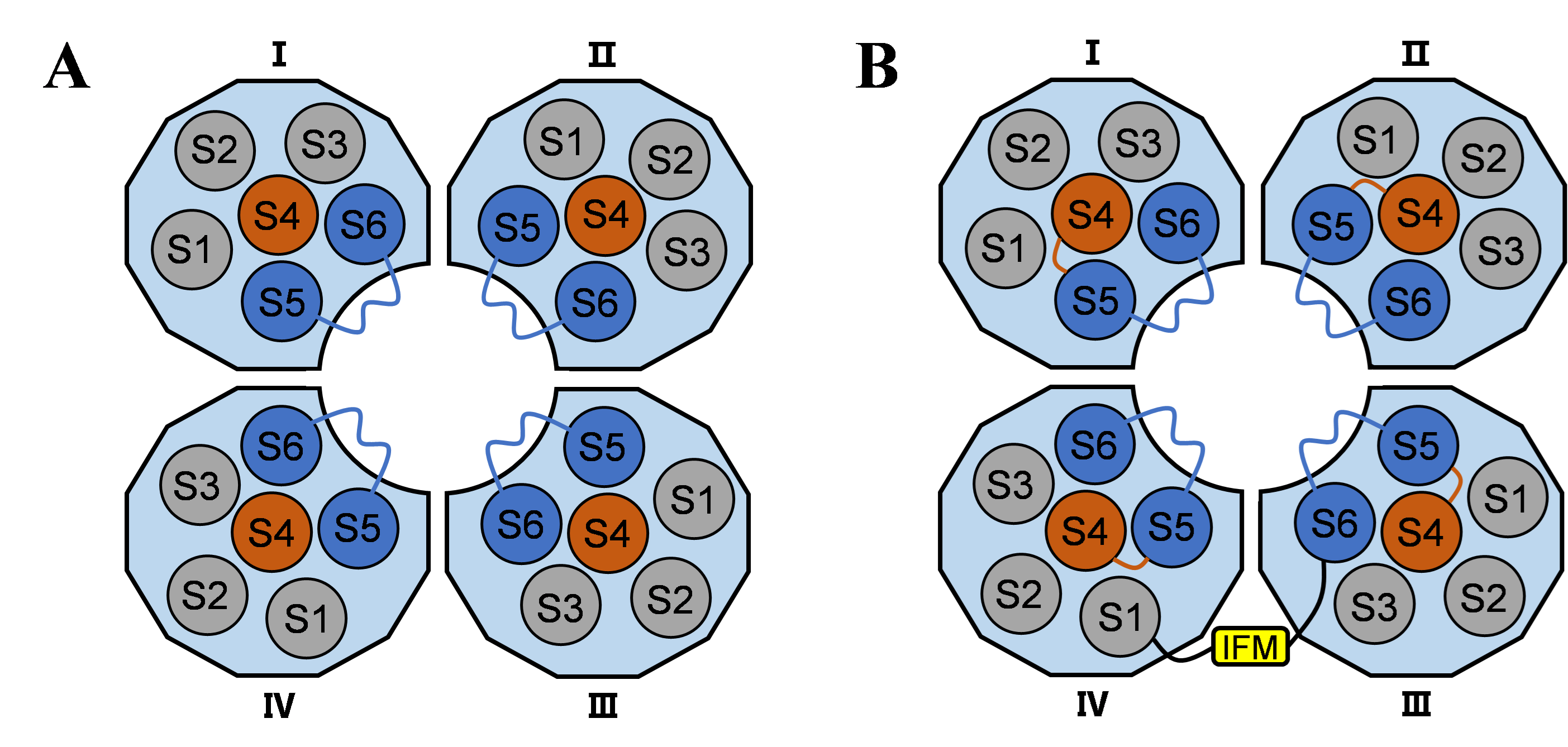

Supplement: Supplementary file 1 [file ijms-27-00732-s001.zip › Fig. S1.tif]

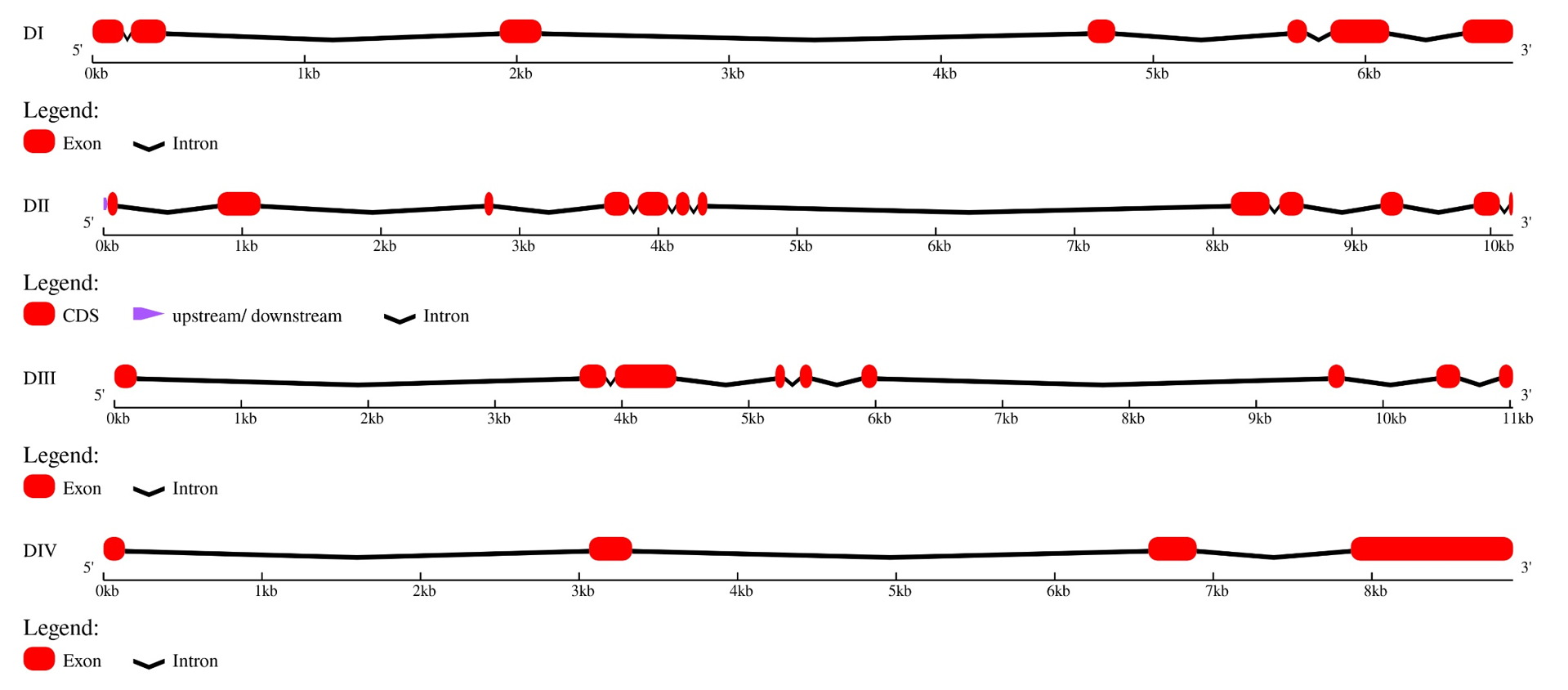

Supplement: Supplementary file 1 [file ijms-27-00732-s001.zip › Fig. S2.tif]

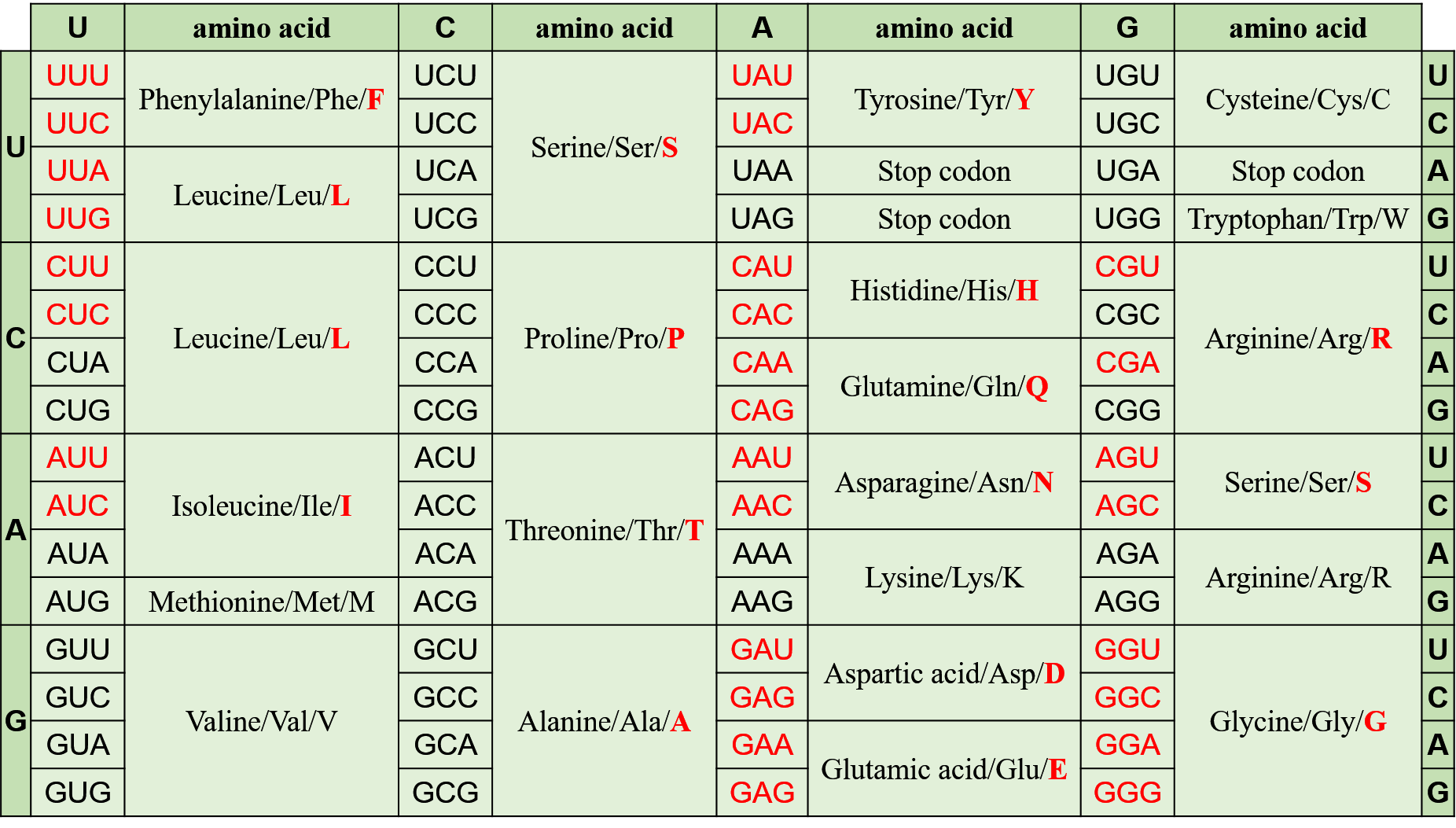

Supplement: Supplementary file 1 [file ijms-27-00732-s001.zip › Fig. S3.tif]
